# *Camelus dromedarius* brucellosis and its public health associated risks in the Afar National Regional State in northeastern Ethiopia

**DOI:** 10.1186/1751-0147-55-89

**Published:** 2013-12-17

**Authors:** Wesinew Adugna Bekele, Tesfaye Sisay Tessema, Simenew Keskes Melaku

**Affiliations:** 1 National Animal Health Diagnostic and Investigation Center, Sebeta, Ethiopia; 2 College of Natural and Computational Sciences, Addis Ababa University, Addis Ababa, Ethiopia; 3 College of Veterinary Medicine and Agriculture, Addis Ababa University, P.O. Box 34, Debre Zeit, Ethiopia; 4 College of Agriculture and Natural Resources, Dilla University, Dilla, Ethiopia

**Keywords:** Brucellosis, *Camelus dromedarius*, CFT, mRBPT, Pastoralists’ risky practices

## Abstract

**Background:**

A cross-sectional study was carried out in four districts of the Afar region in Ethiopia to determine the prevalence of brucellosis in camels, and to identify risky practices that would facilitate the transmission of zoonoses to humans. This study involved testing 461 camels and interviewing 120 livestock owners. The modified Rose Bengal plate test (mRBPT) and complement fixation test (CFT) were used as screening and confirmatory tests, respectively. SPSS 16 was used to analyze the overall prevalence and potential risk factors for seropositivity, using a multivariable logistic regression analysis.

**Results:**

In the camel herds tested, 5.4% had antibodies against *Brucella* species, and the district level seroprevalence ranged from 11.7% to 15.5% in camels. The logistic regression model for camels in a herd size > 20 animals (OR = 2.8; 95% CI: 1.16-6.62) and greater than four years of age (OR = 4.9; 95% CI: 1.45-16.82) showed a higher risk of infection when compared to small herds and those ≤ 4 years old. The questionnaire survey revealed that most respondents did not know about the transmission of zoonotic diseases, and that their practices could potentially facilitate the transmission of zoonotic pathogens.

**Conclusions:**

The results of this study revealed that camel brucellosis is prevalent in the study areas. Therefore, there is a need for implementing control measures and increasing public awareness in the prevention methods of brucellosis.

## Introduction

Brucellosis is considered by the Food and Agriculture Organization of the United Nations (FAO), the World Health Organization (WHO) and Office International des Épizooties (OIE) as one of the most widespread diseases in the world [1]. According to the OIE, it is the second most important zoonotic disease in the world, accounting for the annual occurrence of more than 500,000 human cases [2]. Brucellosis can affect almost all domestic species, and cross transmission can occur between cattle, sheep, goats, camels and other species [3], causing significant reproductive losses in sexually mature animals [4]. The disease is manifested by late term abortions, weak calves, stillbirths, infertility and characterized mainly by placentitis, epididymitis and orchitis. *Brucella melitensis*, *B. abortus* and *B. suis* are zoonotic pathogenic species which can also infect humans. *B. canis* may cause infections in immunosuppressed individuals [5,6].

Globally, this disease is under-reported because of its vague clinical symptoms, difficult laboratory diagnosis and lack of familiarity of the medical professionals [7]. Within Sub-Saharan Africa (SSA), many of the known infectious diseases occur commonly and are poorly controlled, both in livestock and in human populations [8,9]. It has been stated that in SSA, the epidemiology of brucellosis in humans and livestock is not well understood, and available data is limited [1,10].

The Ministry of Agriculture and Rural Development (MoARD) estimates that nationally, pastoralists own almost all of the camels (approximately 1 million head) [11]. Under Ethiopian context, livestock of different species usually share pastures and dwellings. Brucellosis is common in rural areas because farmers live in close contact with their animals and often consume fresh unpasteurized dairy products. However, the vending of dairy products may also bring the disease to urban areas [6,12]. Brucellosis is widely distributed in the region, with small ruminants remaining the most prevalent hosts. Previous work in different districts of the region has revealed a prevalence ranging from 0–11.7% [13]. In camels, the prevalence rate is 4.2% [14] in the South Omo zone of Ethiopia. In the Amibara district of the Afar region, Woldegebriel [15] reported a prevalence rate of 7.6% in camels.

In the region, risky activities, such as the traditional habits of raw milk consumption, handling of aborted materials, manipulation of reproductive excretions with bare hands, and herding of a large number of animals collectively, are widely practiced. As the disease has veterinary, public health and economic importance, it is necessary to assess the current status among camels in selected districts of the Afar region. The objectives of this study were to determine the seroprevalence of brucellosis in camels of selected districts in the Afar region, to investigate associated risk factors for brucellosis in camels, and to assess the awareness level of the Afar pastoral community about zoonotic brucellosis.

## Materials and methods

### Description of study area

The Afar National Regional State (ANRS) is located in the northeastern part of the country. Administratively, the region is divided into five zones, which are further subdivided into 32 districts and 358 peasant associations. The region is organized in such a way that the zone is comprised of several adjacent districts, and peasant associations (PAs) are the smallest governmental administrative sections. Pastoralism and agro-pastoralism are the two major livelihood ways practiced in the region, and according to the official population statistics, the region’s population is estimated to be 1.2 million; of which 90% are pastoralists and 10% agro-pastoralists (a mixture of livestock rearing with rain fed and irrigated crop production). The total surface area of the region is estimated to be 97,970 km^2^[16].

**Figure 1:**
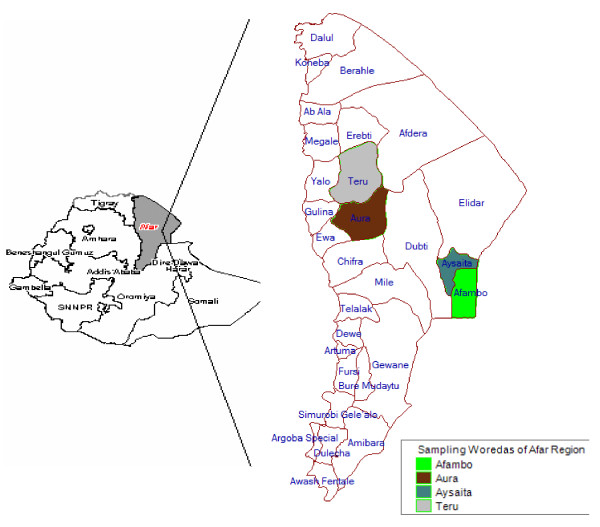
Map of Afar region and the study districts.

The study was conducted in four districts, Afambo, Aysaita, Teru and Aura, which are located in zones one and four (Figure 1). We have selected the study zones, districts, PAs and individual camel owners based on their willingness to participate in the study, and the convenience for our study with respect to transport access and herd mobility scenarios during the study period. Here under is the relevant information for each of the four districts of the region considered for the study.

Aysaita District: A lowland area with an altitude of about 350 meters above sea level (a.s.l.). It has a mean minimum and maximum temperature of 28°C and 45°C, respectively. The district is a destination for pastoralists from zone four and neighboring districts of zone one in search of pasture for their livestock, because the district is endowed with several large-scale irrigation farms using the Awash River, which attracts the pastoralists to feed agricultural leftovers.

Afambo District: This district is characterized by high humidity and temperature, which range between 27°C to 45°C. The district has one urban and eight rural peasant associations (PAs), of which three (Alasa Bolo, Humodoyeta and Mego) are totally agro-pastoralist PAs. The elevation of the district is between 280–850 meters a.s.l., with an average annual temperature of 120 mm. The land coverage of the district is about 1926 km^2^ and the district is known by Lake Abbi, which is the final destination for the Awash River [16].

Teru District: This district is characterized by flat and mountainous landscapes, and is found in the lowland of Afar. The climate is arid with minimum and maximum temperatures of 28°C and 50°C, respectively. This district has two main rainy seasons, Karma (July-Sept) and Sugum (March-April), along with a short rainy season called Dadaa in January. There are dry seasons called Gillal and Hagay. The season of Gillal is relatively colder than the other seasons. The annual rainfall is from 200 mm-800 mm, and there are no perennial rivers. The main water sources are the seasonal rivers, Ela, and ponds. The district is covered by sparse *Acacia* species and extensive grazing land [16].

Aura District: This district covers 3096 km^2^ and it has established eleven kebele centers. The total population of this district is about 28,704 (53% male and 47% female). The principle livelihood of the people is pastoralism, with some PAs practicing agricultural activity. They are in a transition phase into agro-pastoralism. The climate is generally arid to semiarid with a high temperature, and it has a rainy season similar to the Teru district [16].

### Study population

The study population included the camel populations in Awesi Resu (zone one) and Fanteyna Resu (zone four) of the Afar Regional State. The approximate number of camels in Awesi Resu (zone one) was 165,776 [17]; while in Fanteyna Resu (zone four) there were approximately 136,720 head [16]. Camels which were above 6 months of age, with no history of vaccination against brucellosis, were included in this study. The individual animal’s age, sex and herd size were recorded. Then, the camel herd size per household was classified as ≤ 20 and > 20, taking into account the average size of the camel herds in the area. Moreover, based on the age of sexual maturity, the camels were classified into ≤ 4 years and > 4 years.

### Study design

A cross-sectional study design was conducted from November, 2011, until April, 2012, to determine the seroprevalence of *Brucella* infections in camels in selected pastoral and agro-pastoral residences of the Afambo, Aysaita, Aura and Teru Districts, and to identify potential risk factors associated with seropositivity. First, four districts and about 30% of the PAs per district were selected purposively, based on easier accessibility and camel populations. There are 7, 11, 9 and 12 PAs in the Afambo, Aysaita, Aura and Teru districts, respectively. The PA is the lowest administrative unit within a district. Moreover, one-hundred-twenty willingly selected pastoralists were included in the study for a questionnaire survey. Sera were collected from the camels and questionnaires were administered to each randomly selected livestock owner (Table 1).

**Table 1:** Study population and sample size per district

**Zone**	**District**	**Total No of PAs**	**No of PAs selected**	**PAs selected**	Camel population in the districts*	**Animals sampled per district**	**Number of respondents**
1	Afambo	7	4	Alasabolo	1137	60	30
				Mego			
				Humodoyeta			
				Deka			
	Aysaita	11	3	Galifage	13277	151	30
				Henele			
				Urmiytu			
4	Aura	9	3	Deritu	29668	100	30
				Lekoma			
				Mesgid			
	Teru	12	3	Uidolul	29668	150	30
				Asabera			
				Alelo			
	**Total**	**39**	**13**			**461**	**120**

*ARFEB, 2007.

### Sampling method

A multistage sampling technique was used in the survey of the camels, and the PAs were regarded as the primary units, the herds as the secondary units, and the individual animals as the tertiary units. An average of 40% of the camels aged 6 months and above were picked randomly from each selected herd, until the calculated sample size was complete, based on owner willingness. The camel herds in 13 PAs from four districts were sampled during the study, based on the camel population of each district. In order to determine the desired sample size of camels, a lack of previous data on the prevalence of camel brucellosis in the districts was considered. Hence, the average expected prevalence was assumed to be 50% for the area, within 95% Confidence Intervals (CI) at 5% desired accuracy. Subsequently, based on a previous study [18], the sample size (n) was calculated using the formula:

$$n=\frac{1.96^{2}\times P_{\mathrm{ex}}\times \left(1-P_{\mathrm{ex}}\right)}{d^{2}}$$

Where n = sample size, d = desired absolute precision (0.05), P_ex_ = expected prevalence (50%); thus the desired sample size for p = 0.5 is 384 camels. However, it was safer to increase the sample size by two to three-folds as far as practically possible; therefore 461 camels were sampled. Sampling was proportionally distributed based on the total camel population in the study districts and PAs (Table 1). A total of 461 sera samples were collected from 100 herds of camels with no history of previous vaccination against brucellosis.

### Questionnaire survey

**Figure 2:**
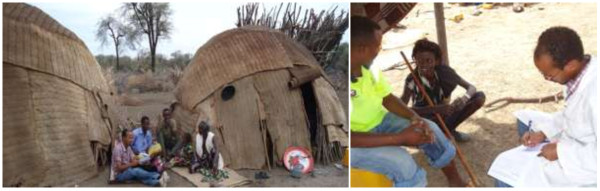
Procedures of questionnaire data collection using local language translator.

Verbal consent was obtained from the respondents, and the objectives of the survey were explained to them before the start of the interview. The interviews were conducted in the local language (Afarigna). Two questionnaire formats, one for the serum sampled individual animal history and the other with a structured questionnaire format for the herders, were developed and used in this study. The questionnaire focused on animal feeding and housing practices, knowledge about zoonotic diseases, the habits of animal product consumption and handling, and dead-animal/aborted fetus disposal practices. In total, 120 pastoralists whose animals were tested for brucellosis were interviewed. In doing so, the risk factors that have possible associations with the occurrence of brucellosis were investigated and used to support the serological results (Figure 2).

### Blood sample collection

Blood samples were collected from the jugular vein of each animal using plain vacutainer tubes. The blood samples were allowed to clot at room temperature; then, the serum was separated from the clotted blood by decanting to other tubes. The separated sera were stored at -20°C until the laboratory testing was performed by using the modified Rose Bengal plate test (mRBPT) and complement fixation test (CFT).

### Serological tests

The modified Rose Bengal plate test (mRBPT) was done in the Semera Regional Veterinary Laboratory in order to screen positive samples by mRBPT, using the mRBPT antigen (Institue Pourquier 325, rue de la galèra 34097 Montpellier cedex 5, France). Positive sera for mRBPT were then retested using the CFT at the National Veterinary Institute (NVI) in Debre Zeit for confirmation. Samples were considered positive for brucellosis if they were positive for mRBPT and CFT on the serial reading basis.

For the mRBPT, the procedure described by Alton *et al*. [19] was followed. The sera and antigen were taken out of the refrigerator and left at room temperature for at least 30 minutes before the test was done. Then, 75 μl of test sera were dispensed on each of the 12 circles of the plate. The antigen bottle was gently shaken and a drop of mRBPT antigen (25 μl) was placed alongside the serum. The antigen and serum were mixed thoroughly using an applicator stick and the plate was rocked manually for about 4 minutes. Finally, agglutination reactions were read in a good light source, or using a magnifying glass when micro-agglutinations were suspected. Reactions were categorized as 0, +, ++ and +++ according to Nielsen and Dunkan [20], where: 0 = no agglutination, + = barely perceptible agglutination (using magnifying glass), ++ = fine agglutination (some clearing), and +++ = clumping, definite clearing. Those samples identified with no agglutination (0) were regarded as negative, while those with +, ++ and +++ were regarded as positive.

Sera positive for the mRBPT were further tested with CFT for confirmation using the standard *Brucella abortus* antigen (New Haw, Addleston, Surrey KT15 3NB, UK). The CFT test proper and reagent preparation procedures followed the procedures outlined by the OIE [21]. A sheep red blood cell (1%) suspension was prepared before the beginning of the test, and test sera with ≥ 50% sedimentation at a dilution of 1:5 and above were considered to be positive. The reading was: complete fixation (no hemolysis) with a water-clear supernatant was recorded as + + + +, nearly complete fixation (75% clearing) as + + +, partial hemolysis (50%) as + + and some fixation (25% clearing) as +. Complete lack of fixation (complete hemolysis) was recorded as 0. For positive reactions, the final titration was recorded.

Sera with strong reactions, more than 75% fixation of the complement (3+) at a dilution of 1:5 or at least with 50% fixation of the complement (2+) at a dilution of 1:10 and above, were classified as positive for the specific CFT [21]. The test was examined by visualization; sedimentation of the sensitized RBCs was considered to be positive and the complete lysis of the sensitized RBCs was taken as negative.

### Data processing and statistical analysis

The data were summarized and individual animal level seroprevalence was calculated on the basis of mRBPT and CFT positive results, divided by the total number of animals tested. Coded data were stored in a Microsoft Excel 2007 spread sheet and transferred to SPSS® Version 16 for statistical analysis. Descriptive and analytical statistics were computed using the software, SPSS® V16. The logistic regression and Chi-square test (*χ*^2^) were employed to determine the association of risk factors with those of seropositivity to the *Brucella* antibody; the degree of association was computed using the Odds ratio (OR) and 95% confidence interval (CI). A test value was considered to be statistically significant when P < 0.05. The OR was used to indicate the degree of risk factor association with the disease occurrence, signified by 95% confidence intervals. The OR is the ratio of the odds of disease occurring among individuals exposed to a variable, and the odds of the disease occurring among individuals not so exposed [18]. Variable reduction was performed by fitting the univariate logistic regression for each covariate, and variables with a p-value > 0.25 were dropped.

## Results

### Seroprevalence of camel brucellosis

At the herd level, a seroprevalence of 24% was found in the camels, and there was no statistically significant difference between the administrative locations (P > 0.05) (Table 2).

**Table 2:** Herd level seroprevalence of camel brucellosis in the study areas

**Zone**	**District**	**Number tested**	**RBPT positive (%)**	**CFT positive (%)**	**Herd level**	
					**Number tested**	**Positive (%)**
One	Afambo	60	5(8.33)	4(6.67)	16	4(25.00)
	Aysaita	151	6(3.97)	5(3.31)	34	5(14.71)
Four	Aura	151	6(3.97)	5(3.31)	34	5(14.71)
	Teru	150	11(7.33)	10(6.67)	31	9(29.03)
**Total**		**461**	**28(6.1)**	**25(5.42)**	**100**	**24(24.0)**

### Univariate logistic regression analysis of risk factors on camel seroprevalence

The univariable logistic regression analysis of the putative risk factors showed a statistically significant difference in the seroprevalence of brucellosis between the camels from herds > 20 animals and those with ≤ 20 head. In addition, animals > 4 years old were at a higher risk of being seropositive for *Brucella* infections than younger ones (Table 3).

**Table 3:** Association of risk factors with the overall individual level seroprevalence of camel brucellosis

**Variables**	**Category**	**No. sampled**	**Complement fixation test**			
			**Positive (%)**	**OR**	**95% CI**	**P-Value**
Zone	4*	211	9(4.3)			
	1	250	16(6.4)	1.532	0.664-3.546	0.316
District	Teru*	60	4(6.7)			
	Afambo	151	5(3.3)	1	0.301-3.321	1
	Aysaita	100	6(6)	2.087	0.695-6.25	0.19
	Aura	150	10(6.7)	1.12	0.393-3.18	0.833
Sex	Male*	51	3(5.9)			
	Female	410	22(5.4)	1.10	0.318-3.82	0.878
Age	≤4 years*	187	3(1.6)			
	>4 years	274	22(8)			
	≤20* animals	265	8(3)	5.354	1.579-18.158	0.007
Herd size	>20 animals	196	17(8.7)	3.05	1.289-7.22	0.011

*Reference category.

### Multivariable stepwise logistic regression analysis of risk factors for *Brucella* reactivity

Only the sex of the animals was omitted, but the age and herd size became evident. Of the different risk factors which appeared significant for the stepwise logistic regression model were the age-groups [camels > 4 years old (OR = 4.935, P < 0.05) as compared to camels ≤ 4 years old].

### Results of questionnaire survey

**Figure 3:**
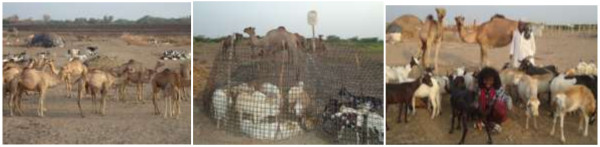
Existing livestock management system in the study areas which allows mixing of small ruminants and camel at different circumstances.

**Figure 4:**
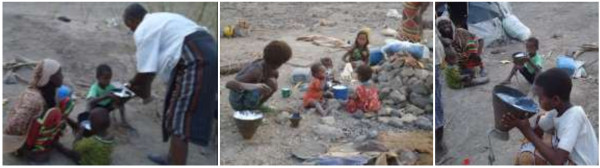
Raw milk consumption habit of the pastoral communities in the study areas.

Very few respondents (30/120 = 25%) agreed with the idea that some diseases can be transmitted from animals to humans. The Afar pastoralists keep different species together at several conditions (Figure 3). One-hundred percent of the respondents consumed raw milk, which contributes to disease transmission. Above three-quarters of the interviewed pastoralists in the study area reported practicing at least one activity considered to be at risk for the transmission of zoonotic diseases (Figure 4).

According to the findings of the questionnaire survey, 61% (74/120), 25.8% (31/120) and 7.5% (9/120) of the respondents keep camels either with both sheep and goats or either of the two or with cattle. Moreover, the mixing of the different species during migration, at watering or in night enclosures (resting), between camels and small ruminants was recorded. More than 75% of the animal owners interviewed did not know about zoonotic brucellosis. In the Afar pastoralist, it is not applicable at all to boil and drink camel milk, and all of the one-hundred twenty respondents said they never boil camel milk. Abortion material and other excreta are handled with bare hands, and they do not destroy these materials. In addition to the above factors they responded about, they also use a common breeding camel bull for different herd groups, although such practices are not that common as most camel owners possess at least one bull in their herd.

## Discussion

In the present study, the seroprevalence of camel brucellosis and associated risk factors, as well as the risky activities practiced by camel herders that may expose them to brucellosis from the camels, was assessed. The overall seroprevalence of brucellosis in this study is in line with the 5.7% seroprevalence in the Afar region reported by Teshome *et al*. [14], and 5.8% by Moustafa *et al*. [22], while it is lower when compared to the 23.8% of Musa *et al*. [23]. Previous data on brucellosis in camels in the same region in the Amibara District found it to be 11.9% by RBPT and 7.6% by CFT [15].

In the Afambo and Aysaita districts, animals are kept in confinement around cultivation fields (more than in the other two districts), because the districts are largely dominated by agricultural irrigation using the Awash River. This may be responsible for the high prevalence in zone one, as infection is easily transmitted within the entire herd under this management system, even if it is not significantly higher. The Teru and Aura districts are mostly pastoralist settings, and are dominated by a free range management system. The use of large grazing sites and river banks as communal grazing areas for camels, and with small ruminant herds, might play a role in the prevalence of the disease.

Brucellosis infection may occur in animals of all age groups, but persists commonly in sexually mature animals. Younger animals tend to be more resistant to infection and frequently clear infections, although a few latent infections may occur [4,24]. Our findings did not conform to the established facts, in that statistically significant differences were observed in the seropositivity between the age groups of camels, being higher in the sexually mature age group. There was no statistically significant difference between the sex groups in the current study. This finding disagrees with the findings of Adamu *et al*. [25] and Junaidu *et al*. [26], both of whom reported a significantly higher seroprevalence of brucellosis among female camels.

Significantly higher seroprevalence was observed in camel herd sizes of > 20 in the current study. The reasons for this significant difference might be attributed to the easy contact among the animals, which favors the bacterial transmission.

Three-fourths of the interviewed pastoralists own mainly sheep, goats and camels, with diverse numbers of each. In addition, the community (most of the time) keeps these species together while browsing, watering, in night enclosures and during migration, which might create an opportunity for the inter-species transmission of the disease. Mohammed *et al*. [27] reported that the seroprevalence of brucellosis in camels in eastern Ethiopia kept without other ruminants, with small ruminants, and with large ruminants was 1.03%, 4.3% and 5.3%, respectively. Moreover, Abou-Eisha [28] observed high seroprevalence in camels with a history of sheep and goats being kept together (with the camels). This may have shown similar results had it been included in this study about the comparison of species seroprevalence.

Factors that contribute to this high prevalence rate in camels may be related to the management system of livestock in the study area. According to the findings of the questionnaire survey, 61% (74/120), 25.8% (31/120) and 7.5% (9/120) of the respondents keep camels either with both small ruminant species or either of the two, or with cattle. Moreover, the mixing of the different species during migration, at watering or in night enclosures (resting) among camels and small ruminants was recorded. Contributing factors to the spread of camel brucellosis may be the movement of animals for grazing and watering, as aggregating the animals around watering points will increase the contact between infected and healthy animals, and thereby facilitate the spread of the disease.

A high number of respondents had no clear knowledge about zoonotic brucellosis. This low awareness is a limiting factor if control strategies are to be implemented, and this may also predispose the community for the disease. Elders are their only source of information, which may indicate paucity in the health education rendered to the community. The most important practices potentially supporting the transmission of zoonotic diseases in the study area were the bare hand management of newborns, aborted fetuses, and fetal membranes, and the consumption of raw milk. Aborted fetuses, though rarely destroyed, are likely to play a role in livestock and human brucellosis. Moreover, the maintenance within the herd, the selling of frequently aborting females to others, and use of a common bull may serve as sources of transmission.

## Conclusion

Brucellosis is prevalent among camels of the study districts, and the risk factors identified for individual animal seroprevalence included age and herd size showing significant differences. Therefore, the disease likely spreads to the unaffected animals and herds, given the extensive production system prevailing in the area, which may allow contact of animals during grazing and at watering points. Moreover, pastoralists are in close contact with their animals, and the consumption of raw milk and handling of aborted materials is common. Thus, there is a need to design and implement control measures aiming at preventing the further spread of the disease in this region.

There is also a need for further study to critically assess the economic impact of this disease, which emanates from its impact on the reproductive and production performance of the animals. Further epidemiological studies and the isolation and identification of the species and biotypes of *Brucella* responsible for infection in the region should be completed. Additionally, detailed studies should be conducted to investigate the link between livestock and human brucellosis, and cross infection between different species.

## Competing interests

None of the authors of this paper have a financial or personal relationship with other people or organizations that could inappropriately influence or bias the content of this paper by any means.

## Authors’ contributions

WA contributed in sample collection, laboratory tests, data acquisition, statistical analysis and drafting of the manuscript. TST involved in the designing of the study, analysis and interpretation of data and write up of the manuscript. SK participated in data collection, analysis and interpretation of data and critical revision of the manuscript. All the three authors read and approved the final version of the manuscript.
